# The Metabolism and Immune Environment in Diffuse Large B-Cell Lymphoma

**DOI:** 10.3390/metabo13060734

**Published:** 2023-06-08

**Authors:** Jianbo Wu, Fuqing Meng, Danyang Ran, Yalong Song, Yunkun Dang, Fan Lai, Longyan Yang, Mi Deng, Yuqin Song, Jun Zhu

**Affiliations:** 1 Key Laboratory of Carcinogenesis and Translational Research (Ministry of Education), Department of Lymphoma, Peking University Cancer Hospital & Institute, Beijing 100142, China; wujb@hsc.pku.edu.cn (J.W.);; 2 Peking University International Cancer Institute, Health Science Center, Peking University, Beijing 100191, China; 3 School of Basic Medical Sciences, Health Science Center, Peking University, Beijing 100191, China; 4 Center for Life Sciences, School of Life Sciences, Yunnan University, Kunming 650500, China; 5 Center for Endocrine Metabolism and Immune Diseases, Beijing Luhe Hospital, Capital Medical University, Beijing 101149, China

**Keywords:** diffuse large B-cell lymphoma, metabolic alteration, metabolic regulation, immune environment, therapeutic strategy

## Abstract

Cells utilize different metabolic processes to maintain their growth and differentiation. Tumor cells have made some metabolic changes to protect themselves from malnutrition. These metabolic alterations affect the tumor microenvironment and macroenvironment. Developing drugs targeting these metabolic alterations could be a good direction. In this review, we briefly introduce metabolic changes/regulations of the tumor macroenvironment and microenvironment and summarize potential drugs targeting the metabolism in diffuse large B-cell lymphoma.

## 1. Introduction

Cells take up various nutrients and undergo different metabolic processes to maintain their growth and differentiation. In normal and tumor cells, they utilize nutrients in different ways. For tumor cells, even when oxygen is present, glucose is still catalyzed by a series of enzymes to generate lactate, rather than being completely oxidized to carbon dioxide, which is called aerobic glycolysis (also known as the Warburg effect). Metabolisms of fatty acids and amino acids have also changed dramatically in tumor cells. These cell-intrinsic metabolic alterations together lead to a highly acidic, nutrient-poor and hypoxic tumor microenvironment (TME), which aggravates the processes of metabolic reprogramming in tumor cells and immune cells within the TME [1]. Ultimately, the metabolic reprogramming accelerates the formation of a tumor-promoting macroenvironment, which is considered a characteristic of cancer [2].

Diffuse large B-cell lymphoma (DLBCL) is aggressive, highly heterogeneous, and the most common form of B-cell lymphoma. Based on cell-of-origin algorithm category and oncogenic mechanisms, DLBCL is classified into three subsets: germinal center B cell-like, activated B cell-like, and unclassifiable subtype [3]. Based on consensus cluster classification with specific metabolic features, DLBCL is classified into three subsets: oxidative phosphorylation (OXPHOS), B-cell receptor/proliferation, and host response subtype [4]. This classification considers more heterogeneous features of DLBCL except for genomics and distinguishes different tumor types from a metabolic perspective [5].

Tumors, including B-cell malignancies, have developed a variety of metabolic strategies to evade antitumor immunity [2]. For instance, DLBCL cells reprogram glucose, amino acid, and fatty acid metabolisms to protect themselves from malnutrition, and subsequently immune cells force themselves to develop metabolic adaptations. However, these metabolic adaptations might reduce the antitumor effectiveness of immune cells [1,6,7,8,9,10]. Developing drugs that regulate cell metabolism could provide a good direction for cancer therapy [11]. In this review, we briefly summarize metabolic alterations/regulations in the DLBCL macroenvironment and microenvironment and potential therapeutic strategies targeting the metabolism in DLBCL.

## 2. Metabolic Alterations of DLBCL Macroenvironment

The concept of “tumor macroenvironment” was introduced by Al-Zoughbi W et al. in 2014 to indicate the pathological interaction among tumor cells, tumor microenvironment and other systems of the body [12]. It is reported that some soluble factors released by tumor cells affect the microenvironment and then reflect on the macroenvironment. Changes in the macroenvironment in turn promote tumor development. Some release soluble factors, such as chemokines, cytokines, or growth factors, which can not only recruit inflammatory cells (such as fibroblasts and myeloid cells) to the microenvironment but also remodel extracellular matrix, initiate and support the formation of new blood vessels, which support tumor growth and lead to more aggressive properties of tumor cells. In addition, new blood vessel networks, nearby tumor cells, and inflammatory cells are imperfect, which leads to accumulation of soluble factors in the tumor microenvironment and then cause pathological endocrine effects and the interaction of the tumor microenvironment with the patient’s organ system [13,14]. Increased levels of inflammatory mediators such as IL-6, TNF-α, IL-1, and IFN-γ are detected in the macroenvironment and are associated with systemic inflammatory responses. Some investigations have demonstrated that the multifactorial in situ network of inflammation controls the complex signaling processes that contribute to tumorigenesis and progression [15,16,17,18]. However, the mechanistic interaction between macroenvironmental factors and tumor development needs to be further explored.

Distinct metabolic features in peripheral plasma were investigated in DLBCL patients with different prognostic outcomes. Mi M et al. collected pretreatment serum samples from 80 DLBCL patients, including germinal center B cell-like (GCB) subtypes and non-GCB subtypes. Then, they tested the serum by the gas chromatography–mass spectrometry (GC-MS) technique, and identified valine, hexadecenoic acid, and pyroglutamic acid as the most important altered metabolites for the prognosis of DLBCL. Higher levels of pyroglutamic acid and hexadecenoic acid in serum are associated with better survival, while increased valine is associated with worse survival [19]. Another team analyzed plasma metabolomics from 22 healthy controls, 25 newly diagnosed DLBCL patients, and 18 complete-remission DLBCL patients. The results demonstrated that compared with complete-remission patients, newly diagnosed patients had decreased levels of glucose and aspartate in the plasma [20].

In addition, several studies have reported that abnormal immunologic markers in autoimmune diseases are significantly associated with the prognosis of DLBCL, which indicates these substances affect disease development through the macroenvironment. Chronic active BCR signaling interacts with several kinase pathways and contributes to DLBCL development, while lack of LYN kinase leads to an autoimmune disease [18,21]. Patients that have high levels of rheumatoid factors, such as anti-double-stranded DNA IgG and anti-nuclear antibody in serum, tend to experience a recurrence of DLBCL [22,23,24]. Abnormal serum immunoglobulins and complements are significantly associated with poor prognosis of patients. For example, there is increased serum IgE level or decreased serum complement C3 level in patients with high International Prognostic Index (IPI) score or high Eastern Cooperative Oncology Group (ECOG) score [25]. Although it remains unclear how tumor cells affect the tumor microenvironment and then change the tumor macroenvironment, this suggests that as tumors expand or disappear, the macroenvironment metabolism changes simultaneously.

## 3. Metabolic Alterations of Tumor Cells in DLBCL Microenvironment

### 3.1. Altered Glucose Metabolism

To meet energy demands, cells utilize glucose-derived carbon through glycolysis or OXPHOS. Although these two metabolic processes occur simultaneously, different types of cells have preferences to use certain metabolic axes. Low-proliferating or highly differentiated cells primarily depend on OXPHOS under aerobic conditions and turn on glycolysis under hypoxia. However, tumor cells, as highly proliferating cells, attain most adenosine triphosphate (ATP) through aerobic glycolysis, even when oxygen is present. This aerobic glycolysis can lead to increased glucose uptake and lactate generation [26]. ^18^F-FDG PET-CT, which is short for 2-deoxy-2-[18F]fluoro-D-glucose positron emission tomography–computed tomography, has been widely utilized in diagnostics and assessment of the therapeutic response in B-cell lymphoma [27]. Several studies have indicated that increased FDG uptake has relationships with expression levels of hexokinase 2 (HK2), glucose transporters (GLUTs) and monocarboxylate transporters (MCTs) in DLBCL tumor cells [28,29].

**Figure 1:**
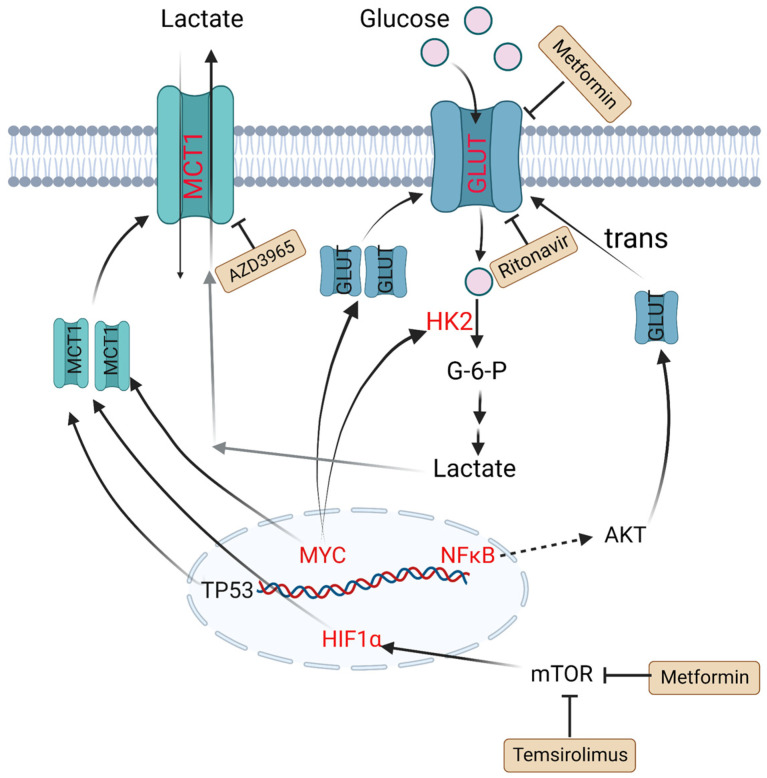
Altered glucose metabolism and potential drugs in DLBCL. Nuclear translocation of NF-κB drives transcription of a gene encoding a scaffold that allows AKT phosphorylation to promote GLUT1 localization to the plasma membrane. MYC promotes lactate production by upregulating HK2 and increases GLUT synthesis. TP53, HIF1α and MYC upregulate the expression level of MCT1. Metformin and temsirolimus are mTOR inhibitors and ritonavir blocks glucose uptake by binding to GLUT4. AZD3965 is an inhibitor of MCT1. Molecules in red represent upregulation in DLBCL. PI3K: phosphoinositide 3-kinase; GLUT: glucose transporter; NF-κB: nuclear factor kappa-B; TP53: tumor protein P53; HIF1α: hypoxia-inducible factor-1α; mTOR: mammalian target of rapamycin; HK2: hexokinase 2; MCT1: monocarboxylate transporter-1.

HK2 is a vital member of the HK family, as the first key rate-limiting enzyme to facilitate glucose to glucose-6-P (G6P) during glycolysis process. It is reported that overexpression of HK2 increases tumor cell glycolysis, and a high level of HK2 is relevant to poor prognosis in DLBCL patients [30]. GLUTs, glucose transporters, can carry and transport glucose across plasma membrane. Their expression and localization are regulated by some factors. For example, nuclear translocation of NF-κB drives transcription of a gene encoding a scaffold that allows AKT phosphorylation to increase GLUT1 localization to the plasma membrane, while tumor protein P53 (TP53), MYC and hypoxia-inducible factor-1α (HIF-1α) upregulate the expression levels of GLUTs and thus lead to greater glycolytic flux in DLBCL [31,32]. MTCs can transfer lactate between different cell types, and are essential for the tumor microenvironment due to high levels of lactate produced by anaerobic glycolysis. Patients with MCT1 upregulation in tumor cells and MCT4 upregulation in stromal cells tend to have poor prognosis. In MYC-amplified lymphomas, MCT1 expression was dramatically increased, which indicates MCT1 expression is regulated by MYC [33,34,35] (Figure 1).

### 3.2. Altered Amino Acid Metabolism

In tumor cells, changes in amino acid metabolism can promote tumor development and progression. For amino acid utilization, tumor cells prefer glutamine as a useful energy source [36]. Glutamine is converted into glutamate by glutaminase (GLS) and finally generates ATP through the tricarboxylic acid (TCA) cycle. It is reported that in many tumor cell lines including B lymphoma, the metabolism of glutamine is modulated by MYC via increasing the expression of GLS. The glutamine transporter protein expression of solute carrier family 1 member 5 (glutamine transporter SLC1A5, also called ASCT2) and solute carrier family 7 member 5 (SLC7A5) can be upregulated by MYC to increase the uptake of glutamine [37]. NAD-dependent deacetylase sirtuin-3 (SIRT3) is a mitochondrial protein deacetylase that stimulates glutamine dissolution by activating GDH to increase α-ketoglutarate (α-KG) production. High expression of SIRT3 in DLBCL tumor cells can replenish the TCA cycle and generate ATP to create biomass, which supports tumor growth [38].

**Figure 2:**
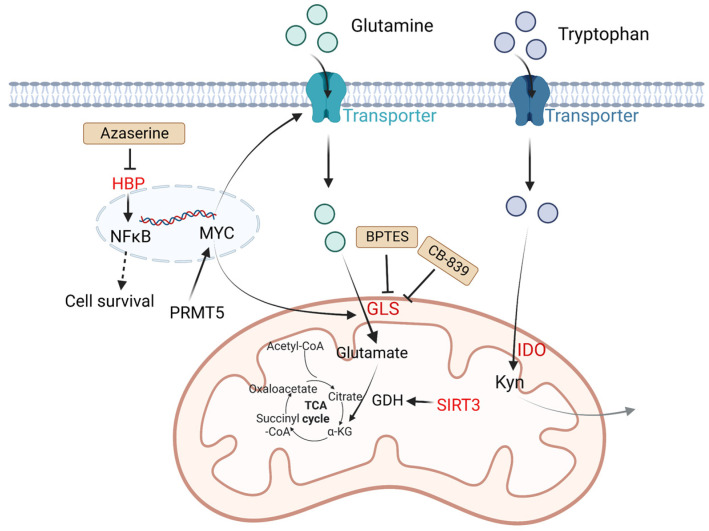
Altered amino acid metabolism and potential drugs in DLBCL. Glutamine is transported by glutamine transporters and then converted into glutamate by GLS. SIRT3 stimulates glutamine dissolution by activating GDH to increase α-KG production, which leads to enhancement of the TCA cycle and thereby generates more ATP to support tumor growth. IDO is a rate-limiting enzyme that catalyzes tryptophan to Kyn and kynurenic acid, which are considered immunosuppressive. PRMT5 promotes transcription of *MYC*. MYC activates GLS and glutamine transporters. HBP activates the NF-κB signaling pathway. The HBP inhibitor azaserine in DLBCL cells can inhibit the activation of NF-κB, and thereby induce cell arrest and apoptosis. BPTES is a selective allosteric regulator of GLS1 that attenuates the growth of lymphoma xenografts and delays MYC-driven tumor proliferation. Another GLS inhibitor, CB-839 inhibits the conversion of glutamine to glutamate. Kyn, catalyzed by IDO1 from tryptophan, is considered immunosuppressive. Molecules in red represent upregulation in DLBCL. GLS: glutaminase; SIRT3: NAD-dependent deacetylase sirtuin-3; PRMT5: protein arginine methyltransferase-5; HBP: the hexosamine biosynthesis pathway; ATP: adenosine triphosphate; TCA: tricarboxylic acid; GDH: glutamate dehydrogenase; α-KG: α-ketoglutarate; Kyn: kynurenine; NF-κB: nuclear factor kappa-B.

Except for glutamine metabolism, other amino acid metabolisms also play important roles in DLBCL. Indoleamine 2,3 dioxygenase (IDO) is a rate-limiting enzyme that catalyzes tryptophan to kynurenine (Kyn) and kynurenic acid [39]. These two products are considered immunosuppressive, since they are agonists of the aryl hydrogen receptor (AHR). AHR induces T_reg_ generation and supports tumor growth by regulating oncogene expression [8]. It is reported that in DLBCL patients, a third of cases have positive IDO expression and worse prognosis compared with IDO-negative DLBCL patients. Sun C et al. found inhibition of IDO1 restrained DLBCL cell proliferation, leading to the upregulation of TP53 in RNA seq analysis [40]. Protein arginine methyltransferase-5 (PRMT5) is an essential enzyme to catalyze dimethylation of arginine on diverse cytoplasmic or nuclear substrates (such as histones) and enhances immunosuppressive function [41]. Erazo T et al. reported that MYC may act as a key target of PRMT5 in B-cell lymphomas [42]. The hexosamine biosynthesis pathway (HBP) requires glucose and glutamine for the O-linked N-acetylglucosamine (O-GlcNAc) cycle, which plays an important role in posttranslational protein modification that adds GlcNAc to nuclear and cytoplasmic proteins. A recent study indicated that the substrates of HBP and O-GlcNAc transferase (OGT) are upregulated in DLBCL tumor cells. Consumption of glucose and glutamine or treatment with an HBP inhibitor (azaserine) in DLBCL cells can reduce the O-GlcNAc substrates and inhibit the activation of NF-κB, further inducing G0/G1 cell arrest and apoptosis [43] (Figure 2).

### 3.3. Altered Fatty Acid Metabolism

To meet increased energy needs, tumor cells reprogram fatty acid metabolism to achieve rapid transformation and progression. The basic source of carbons for fatty acid synthesis in tumor cells comes from glucose. In the mitochondria, glucose is converted into acetyl-CoA then citrate. The mitochondrial citrate transporter protein (CTP) transfers citrate out of the mitochondria. Subsequently, cytosolic citrate and coenzyme A (CoASH) are catalyzed by ATP citrate lyase (ACLY) to oxaloacetate and acetyl-CoA, and the latter is used to synthesize fatty acids [44].

Fatty acid synthase (FASN) is an important enzyme to synthesize 16-carbon fatty acid palmitate, which is highly expressed in more than half of DLBCL samples. Its overexpression is related to poor prognosis. Its inhibitor fasnall is a selective FASN inhibitor that targets multiple domains of FASN, which can restrain c-Met receptor kinase and promote apoptosis of DLBCL cells [45]. The transmembrane protein CD36 functions as the cell surface channel for exogenous fatty acid uptake, which is upregulated and strongly correlated with the proportion of M2 macrophages in CD5^+^ DLBCL. [46].

**Figure 3:**
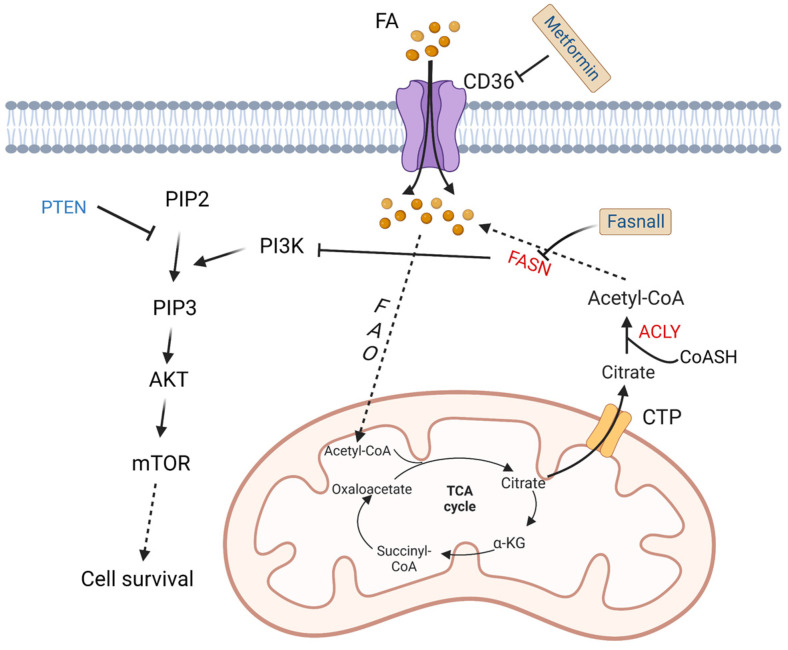
Altered fatty acid metabolism and potential drugs in DLBCL. Fatty acids are transported into DLBCL cells via CD36. Fatty acids are finally converted to acetyl-CoA by a series of oxidation reactions and enter the TCA cycle. A product in the TCA cycle, citrate is transported out of mitochondria by CTP. In cytoplasm, citrate and CoASH are converted to acetyl-CoA catalyzed by ACLY. FASN, the key enzyme in fatty acid synthesis, inhibits the activation of PI3K. PI3K phosphorylates PIP2 to PIP3, and then activates AKT/mTOR pathway to promote cell survival. On the contrary, PTEN dephosphorylates PIP3. Fasnall is a selective FASN inhibitor. Metformin is an inhibitor of CD36. TCA: tricarboxylic acid; FAO: fatty acid oxidation; FASN: fatty acid synthesis; α-KG: α-ketoglutarate; ACLY: ATP-citrate lyase; PYR: pyruvate; CTP: citrate transporter protein; PTEN: phosphatase and tensin homologue; mTOR: metabolic regulator mammalian target of rapamycin; CoASH: coenzyme A. PIP3: phosphatidylinositol 3,4,5-trisphosphate; PIP2: phosphatidylinositol 4,5-bisphosphate.

As a tumor suppressor protein, phosphatase and tensin homologue (PTEN) alterations are found in about 10% of DLBCL cell lines [47], which have a significant opposite relationship with FASN expression in prostate cancer [48]. PTEN dephosphorylates phosphatidylinositol 3,4,5-trisphosphate (PIP3), while phosphoinositide 3-kinase (PI3K) phosphorylates phosphatidylinositol 4,5-bisphosphate (PIP2) to generate PIP3 [49]. Clinically, a majority of *PIK3CA* mutations are found in *PTEN^+^* cases, which indicates a significant correlation between PTEN and the PI3K/AKT signaling pathway [50] (Figure 3).

## 4. Altered Tumor Cell Metabolism Influences Immune Cells in the TME

Tumor metabolic alteration not only plays a crucial role in maintaining tumorigenesis and tumor survival but also has a broader significance in regulating antitumor immune responses through the release of metabolites (such as lactic acid and arginine, etc.). Most of these metabolites impair the function of immune cells [51].

T cells are white blood cells in the immune system and play a central role in adaptive immune response. Based on the presence of cell surface protein CD8 or CD4, they are mainly classified into two major subtypes: CD8^+^ cells and CD4^+^ T cells [52]. Naïve T lymphocytes move through the lymphoid organs, interact with many dendritic cells, and stop when they recognize the specific antigens. Antigen recognition and other activation stimulation induce clonal expansion and differentiation of naïve cells into effector and memory lymphocytes. CD8^+^ effector T (T_eff_) cells are very powerful in the antitumor immune response and can directly kill tumor cells [53]. Unlike CD8^+^ T_eff_ cells, CD4^+^ T helper (Th) cells activate memory B cells or cytotoxic T cells, leading to a larger immune response [52]. Regulatory T (T_reg_) cells, known as inhibitory T cells, express the biomarkers CD4, FOXP3, and CD25 and are thought to be derived from naïve CD4+ cells [54]. T_reg_ cells are immunosuppressive and usually inhibit the proliferation and function of effector T cells [55].

Natural killer cells, also known as NK cells, are cytotoxic lymphocytes that are critical to the innate immune system. As adaptive immune cells, T cells detect the major histocompatibility complex (MHC) on the surface of the tumor cell, inducing the release of cytokines that cause tumor cell death. However, NK cells are unique in that they can recognize and kill tumor cells in the absence of MHCs, allowing for a faster immune response. This role is very important because tumor cells often lack MHC I markers. Therefore, they cannot be detected and destroyed by T lymphocytes, but can be destroyed by NK cells [56].

Myeloid-derived suppressor cells (MDSCs) are a class of highly heterogeneous myeloid cells that develop from myeloid progenitor cells located in the bone marrow. There are very few in the peripheral blood of healthy people, but they are greatly amplified and migrate to the tumor area through the peripheral blood circulation in patients. MDSCs can inhibit both adaptive and innate immunity through a variety of mechanisms, including inhibiting T-cell activation, disabling activated T cells, inhibiting NK cell cytotoxicity, and polarizing macrophages towards tumor progression. High levels of MDSC infiltration in the TME are associated with poor prognosis and treatment resistance of patients [57,58].

Neutrophils are the most abundant type of granulocytes and form an important part of the innate immune system. They are short-lived and mobile, since they can enter tissue parts where other cells/molecules cannot. It has been shown that they can recruit and activate dendritic cells and macrophages [59]. However, with tumor progression, neutrophils undergo an immunogenic “switch” from antitumor to protumor. However, this transient reaction may be context-specific [60].

Macrophages, important innate immune cells, play crucial roles in tissue development, homeostasis, and tissue repair. Based on the function and level of cytokine secretion, macrophages are divided into classically activated (M1) subtype and alternatively activated (M2) subtype [61]. M1 macrophages secrete proinflammatory cytokines to perform antibacterial and antitumor functions. On the contrary, M2 macrophages secrete anti-inflammatory cytokines to perform repair, protumoral and antiparasitic functions [62]. Programmed death ligand 1 (PD-L1) and its receptor are negative indicators of immune cell activation. A study on DLBCL has shown that the expression level of PD-L1 reflects the abundance of activated tumor-infiltrating macrophages, which are associated with the anti-CD20 response. High expression of macrophage (M1) characteristics is also associated with prolonged progression-free survival [63]. It has been reported that oxidative phosphorylation is associated with M1 macrophages, while M2 macrophages depend on glycolysis [64].

**Figure 4:**
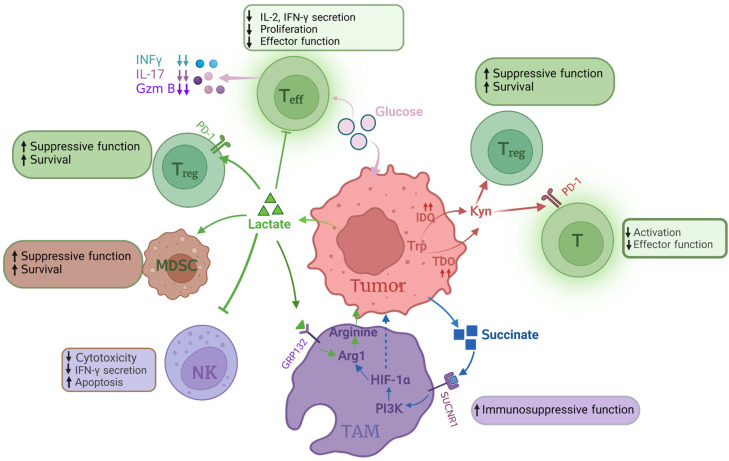
Influence of altered tumor metabolism on immune cells. In the TME, most glucose is consumed by tumor cells, resulting in glucose shortage of T_eff_, which decreases the section of IFN-γ, IL-7 and GzmB. Lactate accumulation in TME influences proliferation, effector function and secretion IL-2 and IFN-γof T_eff_. Lactate inhibits NK cell cytotoxicity, IFN-γ secretion and promotes NK cell apoptosis. Lactate also enhances T_reg_ survival and immunosuppressive function via increasing PD-1 expression. The immunosuppressive function of TAMs is also promoted by lactate via activating Arg1 to produce arginine. Succinate, one of the metabolites in glucose metabolism, is able to enhance TAM immunosuppressive function via the PI3K-HIF-α pathway. IDO catalyzes tryptophan to Kyn, which impacts T_eff_ function and enhances T_reg_ survival. In this figure, the arrow “up” means increase or enhance; “down” means decrease or weaken. T_eff_: effector T cell; T_reg_: regulatory T cell; GzmB: granzyme B; NK: natural kill cell; TAMs: tumor-associated macrophages; Arg1: arginase 1; PI3K: phosphoinositide 3-kinase; HIF1α: hypoxia-inducible factor-1α; IDO: indoleamine 2,3 dioxygenase; TDO: tryptophan 2, 3-dioxygenase; Kyn: kynurenine.

As mentioned earlier, in order to meet their rapid proliferation needs, DLBCL cells undergo some metabolic changes. These metabolic changes influence their cross talk with the immune cells in the TME; therefore, they evade attack from the immune system. In this part, we briefly summarize related studies including other types of tumors, which might provide some clues to explore the effect of altered metabolism on immune cells in the TME of DLBCL (Figure 4, Table 1 and Table 2).

### 4.1. Glucose

B-cell malignancies produce a prominent proportion of ATP by anaerobic glycolysis, and glycolysis produces a lot of lactic acid [28,29,34]. Lactic acid plays important roles in signal transduction within the TME. After sensing lactic acid signals, G protein-coupled receptor GPR132 on macrophage membrane induces the expression of a variety of chemokines and promotes tumor metastasis and progression. Lactic acid uptake promotes the expression of programmed death ligand 1 (PD-L1) in tumor-associated macrophages (TAMs) and MDSCs, and thereby contributes to tumor immunosuppression [65,66]. Olfr78, an odor receptor on lung tumors, collaborates with GRP132 to sense lactic acid signals and upregulate arginase 1, and thereby promotes tumor growth and metastasis [67,68].

Lactic acid has also been shown to enhance the function of T_reg_ cells, inhibit the function and survival of T and NK cells, and lead to tumor immune escape [69,70,71,72]. In a hyper-glycolytic environment, T_reg_ cells have higher PD-1 expression than effector T (T_eff_) cells. When tumor cells consume glucose, T_reg_ cells actively absorb lactic acid via MCT1 to promote nuclear factor of activated T cell (NFAT1) translocation to the nucleus, and thereby enhance PD-1 expression [69]. However, excessive intake of lactic acid in NK cells can cause intracellular acidification and inhibit NFAT signaling in T and NK cells, leading to reduced production of NFAT-regulated interferon-γ (IFN-γ) and promoting apoptosis. In mice with normal immune capacity, reduced production of lactic acid can slow tumorigenicity and significantly increase infiltration of CD8^+^ T cells and IFN-γ-secreting NK cells [73,74].

In the TME, tumor cells have a greater ability to consume glucose, resulting in limited glucose supply to antitumor T cells. Reducing glucose concentration in growth media has been shown to inhibit aerobic glycolysis and increase rates of OXPHOS, leading to attenuated mTOR signaling, and thus inhibit the function of CD4^+^ and CD8^+^ T_eff_ cells [75,76,77]. Decreasing glucose availability in medium inhibit production of many important effector molecules in T_eff_ cells, such as IFN-γ, interleukin 17 (IL17) and granzyme B (GzmB) [76,77,78,79]. What is more, IL6 secreted by mesenchymal fibroblasts increases glycolysis flux in pancreatic tumor cells and lactate efflux in the TME through activation of the signal transducer and activator of transcription (STAT) signaling pathway. Treatment of pancreatic tumors with anti-IL6 antibodies reduces M2 macrophages, increases sensitivity to PD1 therapy, and leads to tumor regression [80].

### 4.2. Amino Acids

Glutamine metabolism plays a key role in tumor cell metabolism. Under nutrient-poor conditions, tumor cells obtain glutamine by breaking down large molecules. For example, activation of the oncogene RAS can promote endocytosis, and tumor cells utilize these extracellular proteins to degrade into amino acids, providing nutrients for themselves [81]. Immune cells highly require glutamine to support cell fate decisions and immune responses. Glutamine deprivation inhibits T-cell proliferation and cytokine production [82,83,84]. However, when T cells are activated, restriction of glutamine has been found to promote memory CD8^+^ T-cell differentiation in vitro [85]. In vivo study has shown that blocking the glutamine pathway in tumor cells increases the level of amino acids in the tumor microenvironment and enhances the cytotoxic effect of immune cells [86].

Most tumor cells lack argininosuccinate synthetase 1 (ASS1), which is a key enzyme that produces arginine [87]. Therefore, tumor cells utilize exogenous arginine to compensate for the lack of arginine within the cell, which leads to less arginine in the TME. Arginine also plays a crucial role in T-cell activation and the regulation of immune response. For example, supplementation of arginine stimulates cytotoxic and effector cytokine production in T and NK cells in vitro, and when combined with anti-PD-L1 antibody therapy, it significantly enhances antitumor immune response and prolongs survival in osteosarcoma mice [88].

Some metabolites of the TCA cycle contribute to the immunosuppressive function of TAMs. Succinate, which is produced by tumor cells, activates the succinate receptor SUCNR1 in TAMs and subsequently stimulates PI3K-HIF-1α axis to promote tumor metastasis [67,89]. Amino acids can also be used as signaling molecules to participate in the regulation of the metabolism in TAMs [90]. Tryptophan is an amino acid necessary for protein synthesis and other metabolic activities, and is ingested by CD98 (consists of heavy-chain SLC3A2 and light-chain SLC7A5) [91].

Tryptophan is degraded to Kyn by two dioxygenases: IDO and tryptophan 2, 3-dioxygenase (TDO) [92,93]. High levels of IDO in tumor cells promote tumor progression and are associated with poor prognosis in patients with gastric adenocarcinoma [94]. IDO contributes to tumor-induced tolerance by inhibiting T-cell function and enhancing local T_reg_-mediated immunosuppression, while TDO also inhibits T-cell-mediated immune responses. Strategies blocking IDO and TDO enhance the effectiveness of tumor immunotherapy [92,93]. What is more, the metabolite Kyn can upregulate the expression level of PD-1 in T cells, enhance T_reg_-mediated immunosuppression and restrict T_eff_ and NK cell response by induction of T-cell exhaustion and deregulation of activation receptors in NK cells [95,96,97,98]. In vitro experiments show that Kyn also leads to upregulation of the PD1 co-inhibitory pathway on activated CD8^+^ T cells. In fact, increased L-kynurenine serum concentration is associated with poor overall survival in DLBCL [99].

### 4.3. Fatty Acids

Tumor cells have a higher rate of de novo fatty acid synthesis, in order to convert energy into anabolic pathways and then produce plasma membrane phospholipids and signaling molecules. Fatty acid synthesis provides cell membranes and other key lipid cell structures for immune cell proliferation. In tumor-infiltrating myeloid cells (such as MDSCs and TAMs), the abnormal accumulation of lipid metabolites (such as short-chain fatty acids, long-chain fatty acids, cholesterol, etc.), has been shown to tilt these immune cells towards immunosuppressive and anti-inflammatory phenotypes through metabolic reprogramming [100]. Emerging evidence indicates the importance of cholesterol metabolism in innate immune responses. High cholesterol levels caused by tumor cells can promote the expression of T-cell-inhibitory immune checkpoints (such as PD-1, LAG-3 and TIM-3), and thereby deprive their antitumor effect [101]. It has been found that the transcription factor STAT1, a key regulator of cholesterol metabolism and the arachidonic acid pathway, is significantly correlated with the proportion of some specific T cells and M1 macrophages, suggesting its role in regulation of the TME [102].

Arachidonic acid is an essential fatty acid in tumor cells and is an important substrate for the synthesis of prostaglandins. Prostaglandin E_2_ (PGE_2_) is a crucial cell growth and regulation factor that also plays important roles in regulation of immune responses [103]. Cancer-associated fibroblast-derived PGE_2_ or other sources of PGE_2_ can induce cancer cell invasion and participate in tumor progression by stimulating angiogenesis, cell invasion and metastasis, and promote cell survival by inhibiting apoptosis [104]. PGE_2_ secreted by tumor cells can stimulate the secretion of cancer-promoting CXCL1, IL-6 and granulocyte-colony-stimulating factor (G-CSF) by bone marrow-like cells, inhibit the activation of type I interferon-dependent innate immune cells, inhibit T cells from targeting tumor antigens, and transform M1 macrophages with antitumor effect into M2 macrophages with cancer-promoting effect to achieve immune escape [105,106]. PGE_2_ can also exert anti-inflammatory effects on neutrophils, monocytes, NK cells and other natural immune cells, such as inhibiting Th1 differentiation, B-cell function, T-cell activation and anaphylaxis [107,108,109].

In hypoglycemic and hypoxic areas of a mouse melanoma model, CD8^+^ tumor-infiltrating lymphocytes (TILs) enhance peroxisome proliferator-activated receptor (PPAR)-α signal transduction and fatty acid catabolism to maintain their effector functions. Stimulating the fatty acid catabolism of CD8^+^ TILs can improve the immunotherapy effect of melanoma [110], while fatty acid accumulation of CD8^+^ TILs leads to cell dysfunction. For example, in the lipid-rich TME region of pancreatic ductal adenocarcinoma, CD8^+^ T cells decrease the expression of very-long-chain acyl-CoA dehydrogenase (VLCAD) and therefore lead to the accumulation of long-chain fatty acid (LCFA), and ultimately impair mitochondrial function, leading to cell dysfunction [111].

## 5. Potential Therapeutic Strategies Targeting Metabolism in DLBCL

As a standard first-line treatment strategy for DLBCL patients, R-CHOP has a good outcome. R-CHOP responders may vary from 95% (limited stage) to 85% (advanced stage) for a 5-year overall survival rate, and more than 60% of DLBCL patients can be cured with R-CHOP. Patients who fail treatment after R-CHOP usually have poor prognosis [3]. Therefore, explorations of new therapeutic strategies need to be carried out. Several treatments for relapsed/refractory DLBCL involving immunotherapy and molecular pathway inhibitors have shown promising results in DLBCL patients. These treatments include CAR T-cell therapy targeting CD19 [112,113,114], CD19 monoclonal antibody (tafasitamab) [115], CD19 antibody conjugate (loncastuximab tesirine) [116], CD37 antibody conjugate (naratuximab emtansine) [117], CD79b antibody conjugate (Pola) [118,119], and CD3-CD20 bispecific antibody (epcoritamab and Mosun) [120]. Significantly, researchers have also developed a universal CD19–CD22 dual-targeted CAR-T cell therapy, in which the *TRAC* region and *CD52* gene were disrupted through the CRISPR-Cas9 method, in order to avoid host immune-mediated rejection [121]. Zhang J et al. developed a non-viral CAR-T type of anti-CD19 CAR-T cell with PD1 integration through CRISPR-Cas9. In a preclinical study of eight patients with relapsed/refractory aggressive B-cell non-Hodgkin lymphoma, it achieved high safety without serious adverse events and 87.5% complete remission [122]. These strategies offer very promising prospects in the treatment of DLBCL. However, most of these strategies are designed to target surface antigens, which may be lost during the treatment. As DLBCL cells reprogram energy metabolism during rapid cell division, combinations of drugs targeting metabolic pathways with other regimens could improve treatment outcomes.

Glycolytic metabolism is significantly upregulated in DLBCL patients. Ritonavir blocks glucose uptake by binding to GLUT4, and the combination of metformin and ritonavir reduces GLUT4 expression in primary chronic lymphocytic leukemia patients [123]. In a small study, three out of four patients with refractory DLBCL achieved complete responses when treated with an mTOR inhibitor temsirolimus, glutamine inhibitor L-asparagase and metformin combination [124]. As the degree of TME acidification increases, lactic acid is converted to pyruvate to fuel OXPHOS. AZD3965 is an effective inhibitor of MCT1, which inhibits lactic acid efflux and the growth of tumor cells. Combination of AZD3965 and rituximab has shown significant antitumor efficacy compared with rituximab alone [125]. HK2 can inhibit mitochondria-mediated apoptosis, increases anaerobic glycolysis, and therefore becomes another potential therapeutic target (Figure 1, Table 3).

DLBCL cells reprogram amino acid metabolism to meet energy requirements. BPTES is a selective allosteric regulator of GLS1 that attenuates the growth of lymphoma xenografts and delays *Myc*-driven tumor proliferation [126]. In addition, BPTES blocks glutaminolysis and reduces PD-L1 expression of ABC-DLBCL, participating in the immune escape [127]. Another GLS inhibitor, CB-839 inhibits the conversion of glutamine to glutamate and thereby restricts tumor proliferation in non-small-cell lung cancer [128]. Combined utilization of CB-839 with mTOR inhibitors has shown good results in many cancers [129]. As mentioned in Section 3, HBP requires glucose and glutamine for the O-linked N-acetylglucosamine (O-GlcNAc) cycle. Consumption of glucose and glutamine or treatment with an HBP inhibitor (azaserine) in DLBCL cells can reduce the O-GlcNAc substrates and inhibit the activation of NF-κB, further inducing G0/G1 cell arrest and apoptosis [43] (Figure 2, Table 3).

Lipid reprogramming has an important role in DLBCL. FASN is an important enzyme to synthesize 16-carbon fatty acid palmitate. Fasnall is a selective FASN inhibitor that targets multiple domains of FASN, which is able to inhibit the expression of FASN in DLBCL cells [130,131]. The fatty acid transporter CD36 mediates fatty acid metabolism and promotes cancer progression and metastasis. Metformin not only regulates glucose metabolism but also has an effect on fatty acid metabolism. It is reported that addition of metformin dramatically reduces the expression level of CD36 and proportion of M2 macrophages in a co-culture system of DLBCL [46] (Figure 3, Table 3).

Except for drugs, calorie restriction can be another alternative way to improve patients’ prognosis. The results of a small study showed that compared with six participants in the control group, six participants who completed multiple short-term calorie reduction had beneficial hematological parameters after chemotherapy [132]. However, more randomized trials are needed to confirm the effects.

**Table 3:** Structures of potential therapeutic drugs targeting metabolism in DLBCL.

Drug	Structure	Reference
Azaserine	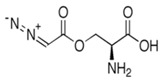	[133]
AZD3965	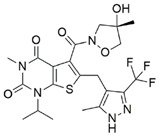	[134]
BPTES	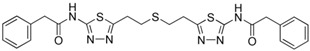	[135]
CB-839	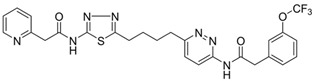	[136]
Fasnall	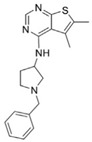	[137]
Metformin	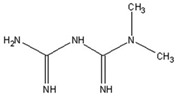	[138]
Ritonavir	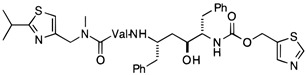	[139]
Temsirolimus	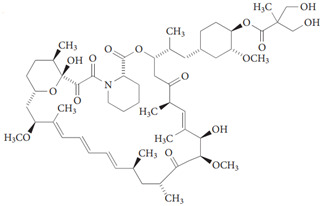	[140]

## 6. Summary

In conclusion, B-cell malignancies have developed a variety of metabolic strategies to protect themselves from malnutrition. These tumor cell-intrinsic metabolic alterations together suppress the function of antitumor immune cells and contribute to the formation of a tumor-promoting microenvironment and macroenvironment. Understanding and utilizing metabolic cross talk of tumor cells and immune cells has the potential to improve the response rate of immunotherapy. On the one hand, these metabolic markers have potential to be therapeutic drugs. On the other hand, we need to consider whether the prognosis of DLBCL could be evaluated through metabolites in serum or urine. It is reported that in urine and serum samples from patients with COVID-19 and healthy controls, the relative abundance of 301 proteins showed opposite expression patterns in urine and serum as the disease progressed [141]. Serum proteins also vary in DLBCL patients with different prognoses, as mentioned in Section 2.

Moreover, metabolic alterations are not unique to B-cell lymphoma, but also are characteristics of other rapidly proliferating cells, including proliferating immune cells. Therefore, scientists need to find more tumor-specific substances to design drugs in order to improve targeting and reduce toxic side effects.

## Figures and Tables

**Table 1:** Tumor-supporting roles in the TME.

Tumor-Supporting Roles in the TME	
**Immune Cells**	**Main Functions**
T_reg_	Inhibit the proliferation and function of T_eff_ cells.
MDSC	Inhibit both adaptive and innate immunity by inhibiting T-cell activation, disabling activated T cells, inhibiting NK cell cytotoxicity, and polarizing macrophages towards tumor progression.
Neutrophil	Undergo an immunogenic “switch” from antitumor to protumor with tumor progression. This transient reaction may be context-specific.
M2 macrophage	Secrete anti-inflammatory cytokines to perform protumoral function.
**Soluble substances**	**Main Functions**
Lactate	Promote tumor growth and metastasis; enhance the function of T_reg_ cells; Inhibit the function and survival of T and NK cells
Glucose	Promote tumor cell growth.
Glutamine	Promote tumor cell growth.
Arginine	Promote tumor cell growth.
Succinate	promote tumor metastasis.
Kynurenine	Upregulate the expression level of PD-1 in T cells; enhance T_reg_-mediated immunosuppression and restrict the T_eff_ and NK cell response by inducing T-cell exhaustion and deregulating activation receptors in NK cell.
Cholesterol	Promote the expression of T-cell-inhibitory immune checkpoints (such as PD-1, LAG-3 and TIM-3).
PGE_2_	Stimulate the secretion of cancer-promoting CXCL1, IL-6 and G-CSF by bone marrow-like cells; inhibit the activation of type I interferon-dependent innate immune cells; inhibit T cells from targeting tumor antigens; transform M1 macrophages into M2 macrophages.

**Table 2:** Tumor-suppressing roles in the TME.

Tumor-Suppressing Roles in the TME	
**Immune Cells**	**Main Functions**
T_eff_	Kill tumor cells by detecting MHC on the surface of the tumor cell and inducing the release of cytokines that cause tumor cell death. These functions are inhibited under limited glucose, or glutamine supply.
Th	Activate memory B cells or cytotoxic T cells.
NK	Recognize and kill tumor cells in the absence of MHCs.
Neutrophil	Recruit and activate dendritic cells and macrophages.
M1 macrophage	Secrete proinflammatory cytokines.
**Soluble substances**	**Main Functions**
Glucose	Promote the function of CD4^+^ and CD8^+^ T_eff_ cells.
Glutamine	Promote T-cell proliferation and cytokine production.
Arginine	Promote T-cell activation.
